# Two new parasitic copepods of the family Sphyriidae (Copepoda: Siphonostomatoida) from Australian elasmobranchs

**DOI:** 10.1007/s11230-022-10054-4

**Published:** 2022-07-17

**Authors:** Geoff A. Boxshall, Diane P. Barton, Amy Kirke, Xiaocheng Zhu, Grant Johnson

**Affiliations:** 1 Department of Life Sciences, Natural History Museum, Cromwell Road, London, SW7 5BD UK; 2 School of Agricultural, Environmental & Veterinary Sciences, Charles Sturt University, Wagga Wagga, NSW 2678 Australia; 3 Research Institute of Environment and Livelihood, Charles Darwin University, Darwin, NT 0801 Australia; 4 New South Wales Department of Primary Industries, Wagga Wagga Agricultural Institute, Wagga Wagga, NSW 2678 Australia; 5 Fisheries Research Division, Northern Territory Department of Industry, Tourism and Trade, Darwin, NT 0801 Australia

## Abstract

Two new species of the genus *Tripaphylus* Richiardi in Anonymous, 1878 (family Sphyriidae) are described from elasmobranch hosts caught as bycatch within the Demersal and Timor Reef Fisheries which operate in the Northern Territory exclusive economic zone. *Tripaphylus squidwardi* **n. sp.** was collected from *Carcharhinus coatesi* Whitley and had a prevalence of 11.6%. *Tripaphylus dippenaarae* **n. sp.** was collected from *Rhizoprionodon acutus* (Rüppell) and had a prevalence of 28.2%. The new species are distinguished from existing congeneric species by the body proportions and shape of the adult female and by the arrangement of lobes on the ventral surface of the cephalothorax.

## Introduction

The copepod family Sphyriidae comprises 42 valid species classified in eight genera (Walter & Boxshall, 2022). The adult females of sphyriids are large, highly transformed parasites that live embedded in the tissues of their marine fish hosts. In contrast, the males are reduced in size and the few that have been reported were found attached around the genital region of the females. Four of the genera and 26 species are reported to utilize actinopterygian hosts with the remaining 16 species from the other four genera found on elasmobranch hosts. The Australian fauna currently comprises just three species. *Sphyrion laevigatum* (Quoy & Gaimard, 1824) was the first to be reported from Australian waters. It was recorded under the name *Sphyrion australicus* Thor, 1900, and was considered to be a new species by Thor (1900). Thor (1900) was unable to provide reliable host data, noting only that the host data accompanying his specimen, caught in 1864, referred to the host only as “la grande morue rouge d’Australie”. Ho (1992) listed *S. australicus* as a synonym of *S. laevigatum* and this remains currently the only species of *Sphyrion* reported from Australia. The same parasite was also reported from New Zealand waters by Thomson (1890) as a new species under the name *Lesteira kroyeri* Thomson, 1890, from the pink cusk-eel *Genypterus blacodes* (Forster).

The other two sphyriids currently known from Australian waters both belong to the genus *Tripaphylus* Richiardi in Anonymous, 1878 and both occur on elasmobranch hosts. *Tripaphylus australis* (Kabata, 1993) was originally described from *Glaucostegus typus* (Anonymous [Bennett]) (Kabata, 1993) caught in Moreton Bay, Queensland, and was subsequently reported from a second host *Aptychotrema rostrata* (Shaw & Nodder) in southeastern Queensland (Turner et al., 2003). *Tripaphylus asymboli* (Turner, Kyne & Bennett, 2003) was described from two species of *Asymbolus*, *A. analis* (Ogilby) and *A. rubiginosus* Last, Gomon & Gledhill, caught off southeastern Queensland (Turner et al., 2003). Both these species were originally placed in the genus *Paeon* Wilson, 1919 but Benz & Boxshall (2017) showed that *Paeon* was a junior subjective synonym of *Tripaphylus* and transferred both species to the latter genus. Dippenaar (2018), in her revision of the genus *Tripaphylus*, accepted 13 species as valid and provided a key to species.

Here we describe two new species of *Tripaphylus* collected from elasmobranch hosts caught in northern Australian waters. A total of 233 *Carcharhinus coatesi* and 39 *Rhizoprionodon acutus* were collected from 9 May 2018 to 8 Nov 2019 as bycatch within the Offshore Snapper Fishery within the Northern Territory exclusive economic zone, in northern Australia. Sharks were stored in brine or frozen whole at the time of capture on the boats; all specimens were subsequently stored frozen until processing. At the time of processing, the head of the shark was removed posterior to the gill region. The gills were then individually examined for the presence of female *Tripaphylus*, as revealed by the protruding trunk. When found, attempts were made to dissect out the head and neck of the copepod from the tissues of the shark host.

## Materials and Methods

The description of the new *Tripaphylus* species from *Carcharhinus coatesi* Whitley is based on the examination of 26 specimens (11 complete females and 15 incomplete females, mostly headless) collected from the ventral throat region of the host. These specimens constitute the type series. A further 20 non-type specimens of *Tripaphylus* were collected as part of the project. The total of 46 *Tripaphylus* specimens came from 27 infected hosts (11 female, 16 male) with a mean total length of 707.8 mm: the prevalence was 11.6%, the mean intensity was 1.7 (ranging from 1 to 6 per host).

This description of the new *Tripaphylus* species from *Rhizoprionodon acutus* (Rüppell) is based on the examination of 4 specimens (2 complete and 2 headless females), collected from the ventral throat region of the host. These specimens constitute the type series. A further 7 non-type specimens of *Tripaphylus* were collected as part of the project. The total of 11 *Tripaphylus* specimens came from 11 infected hosts (7 female, 4 male) with a mean total length of 755.8 mm: the prevalence was 28.2%, the mean intensity was 1.0.

Specimens were cleared in lactic acid and observed whole on a Leitz dissecting microscope. Dissected appendages were examined on an Olympus BH2 microscope using differential interference contrast. Clotted host blood was present on the appendages of most specimens obscuring detail. Drawings were made using a drawing tube and measurements were made using a stage micrometer. Morphological terminology conforms to Kabata (1979) and Huys & Boxshall (1991). In adult females the neck – trunk junction was often difficult to determine with precision but was here identified as the point where the expansion of trunk can first be detected, although in some specimens the expansion is so gradual that the location of the junction should be viewed as approximate. Names of hosts follow FishBase (Froese & Pauly, 2021).

Two specimens collected from *C. coatesi* and one from *R. acutus* were examined using scanning electron microscopy (SEM). They were washed and dehydrated overnight in a series of graded ethanol solutions (Shamsi et al., 2019). After three additional overnight washes in absolute ethanol, the specimens were critical point dried using a Tousimis Autosamdri-931 (USA). Samples were then mounted on a 12 mm carbon tab (ProSci Tech, Australia) and sputter coated with gold using a K550X Sputter Coater (Quorum Technologies, UK). The specimens were examined under a JCM-5000 Benchtop SEM NeoScope (JEOL Ltd. Peabody, Massachusetts, USA) with accelerating voltage set at 10-15kv.

Holotypes of both species are deposited in the Museum and Art Gallery, Northern Territory (MAGNT), paratypes are deposited in MAGNT and in the Natural History Museum, London (NHM),

## Systematics

Family Sphyriidae

Genus *Tripaphylus* Richiardi in Anonymous, 1878

***Tripaphylus squidwardi*** **new species**

Type Material.

Holotype female, intact with one missing egg sac, from *Carcharhinus coatesi* Whitley, 1939 (CARCOA 400, collected 27 Nov 2018; -10.23, 134.65; Male 694 mm TL), MAGNT Registration Number NTM Cr019479; paratype intact female and 2 headless paratype females from same host (CARCOA 400), NTM Cr0179480; paratype female intact but lacking egg sacs, from *C. coatesi* (CARCOA 140, collected 14 May 2019; -10.266, 136.3096; Male 772 mm TL), NTM Cr0179481; paratype female intact but lacking egg sacs, from *C. coatesi* (CARCOA 145, collected 14 May 2019; -10.266, 136.3096; Female 793 mm TL), NTM Cr0179482; 2 paratype females intact and bearing egg sacs, from *C. coatesi* (CARCOA 210, collected 20 Nov 2018; -10.17, 134.65; Male 674 mm TL), NTM Cr0179483; 4 paratype females intact, 3 bearing egg sacs, and 3 incomplete paratype females from *C. coatesi* (CARCOA 632, collected 06 Jun 2019; -8.25, 129.15; Male 645 mm TL), NTM Cr0179484; paratype female intact with egg sacs, from *C. coatesi* (CARCOA 633, collected 06 Jun 2019; -8.25, 129.15; Female 633 mm TL), NTM Cr0179485; paratype female intact with egg sacs, from *C. coatesi* (CARCOA 664, collected 27 Nov 2018; -10.23, 134.65; Male 673 mm TL), NTM Cr0179486; headless paratype female, trunk bearing paired egg sacs, from *C. coatesi* (CARCOA 113, collected 01 Dec 2018; -10.70, 128.73; Male 579 mm TL), NTM Cr0179487; headless paratype female, trunk lacking egg sacs, from *C. coatesi* (CARCOA 136, collected 14 May 2019; -10.266, 136.3096; Female, TL not measured), NTM Cr0179488; 3 headless paratype females, 1 ovigerous and 2 with trunk lacking egg sacs, from *C. coatesi* (CARCOA 149, collected 29 May 2019; -12.5243, 128.5889; Female 743 mm TL), NTM Cr0179489; headless paratype female, trunk with broken egg sacs, from *C. coatesi* (CARCOA 160, collected 28 May 2019; -12.5531, 128.5595; Male 683 mm TL), NTM Cr0179490; headless paratype female, trunk with broken egg sacs, from *C. coatesi* (CARCOA 275, collected 06 Jun 2019; -8.25, 129.15; Female 736 mm TL), NTM Cr0179491; 2 isolated paratype heads plus 3 headless trunks lacking egg sacs, from *C. coatesi* (CARCOA 276, collected 06 Nov 2018; -10.28333, 135.6667; Male 741 mm TL), NTM Cr0179492; headless paratype female, trunk lacking egg sacs, from *C. coatesi* (CARCOA 362, collected 15 Feb 2019; -10.11667, 136.3167; Female 776 mm TL), NTM Cr0179493; headless paratype female, trunk lacking egg sacs, from *C. coatesi* (CARCOA 415, collected 28 Nov 2018; -10.25, 134.13; Female, 645 mm TL), NTM Cr0179494; headless paratype female, trunk lacking egg sacs, from *C. coatesi* (CARCOA 660, collected 28 Nov 2018; -10.25, 134.13; Male 716 mm TL), NTM Cr0179495; paratype female with head intact but trunk incomplete, from *C. coatesi* (CARCOA 670, collected 28 Nov 2018; -10.25, 134.13; Male 731 mm TL), NTM Cr0179496: 4 paratype females intact, 3 bearing egg sacs, and 3 incomplete paratype females from *C. coatesi* (CARCOA 632, collected 06 Jun 2019; -8.25, 129.15; Male 645 mm TL) NHMUK 2022.174-177.

LSID urn:lsid:zoobank.org:act:4836F799-E8B6-4C46-A2CB-8E31F8FF854D

### Description (Figs 1, 2)

**Fig. 1:**
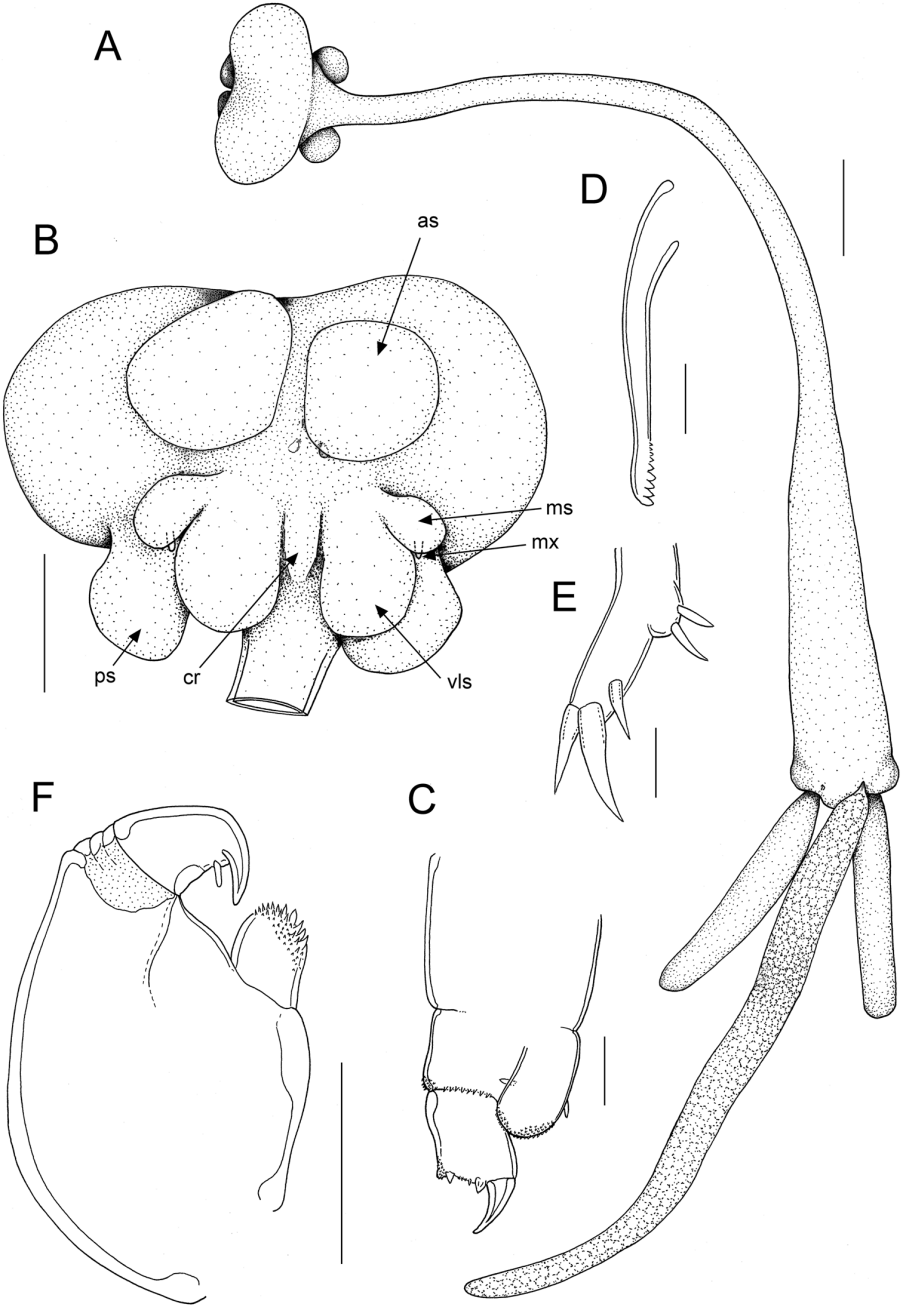
*Tripaphylus squidwardi* **n. sp.** adult female. A, habitus, dorsal; B, cephalothorax, ventral; C, antenna; D, mandible; E, maxillule; F, maxilliped. Scale bars: A, 2 mm, B, 1 mm, C-E, 20 μm, F, 100 μm. Abbreviations: as = antennary swelling, cr = central ridge, ms = maxillary swelling, mx = maxilla, ps = posterior swelling, vls = ventrolateral swelling

**Fig. 2:**
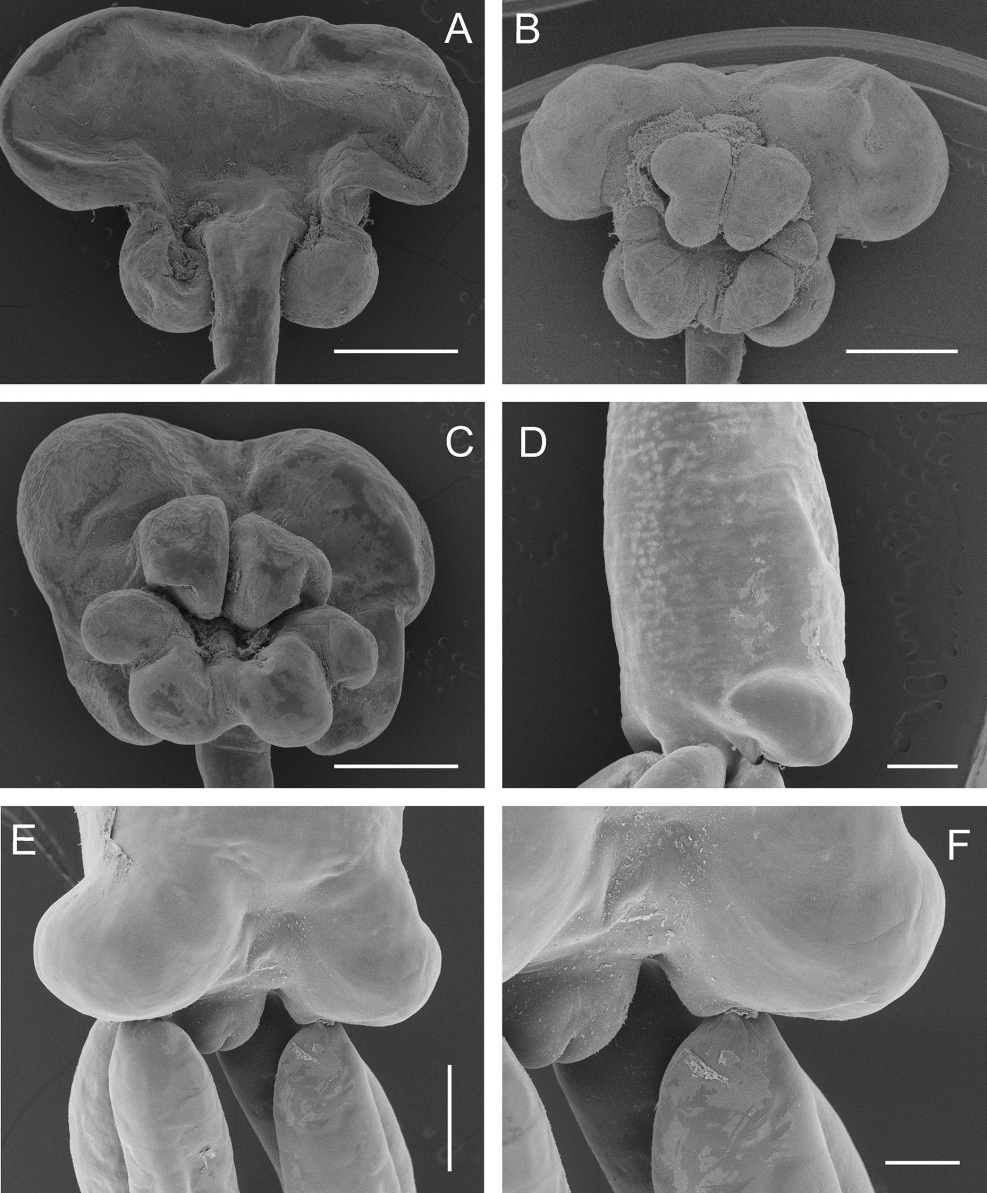
Scanning electron micrographs of *Tripaphylus squidwardi* **n. sp.** adult female. A, cephalothorax, dorsal; B, cephalothorax, ventral; C, cephalothorax of another specimen, ventral; D, Posterior extremity of trunk, lateral view showing lateral lobe at posterior end of trunk; E, Posterior extremity of trunk, showing origins of posterior processes, egg sacs and abdomen; F, abdomen, showing anal prominence. Scale bars: A-C, 1.0 mm, D-E, 500 μm, F, 200 μm

Transformed adult female. Body (Fig. 1A) comprising cephalothorax, neck and trunk incorporating minute abdomen and bearing large paired posterior processes. Measurements of holotype female: total length measured from frontal margin of cephalothorax to posterior margin of anal prominence (excluding posterior processes) 24.5 mm; cephalothorax length 1.6 mm, width 3.9 mm; neck length about 13.8 mm, trunk length about 9.1 mm [neck + trunk combined length 22.9 mm], maximum trunk width 2.1 mm; posterior processes length 4.9 mm, width 1.1 mm; egg sac length 14.4 mm. Mean total body length of intact paratypes 25.1 mm, range 22.3 to 27.3 mm (n = 10).

Neck slender, cylindrical with smooth margin; expanded anteriorly at junction with cephalothorax; expanding gradually posteriorly to merge with trunk. Neck always longer than trunk, mean 1.48 times, with a range of 1.02 to 1.90 times longer (n = 11). Trunk about 4.3 times longer than wide, slightly compressed dorsoventrally and increasing in width towards maximum at posterior end; bearing paired lateral swellings near posterior end (Fig. 2D,E). Abdomen (Fig. 2F) incorporated into trunk. Trunk bearing paired posterior processes ventrally; each process cylindrical, blunt tipped, and ranging from 4.5 to 6.9 times longer than wide, with mean of 5.6 times (n = 13). Egg sacs multiseriate, originating from dorsally located oviduct openings near posterior margin of trunk; typically about twice as long as posterior processes, ranging from 1.8 to 2.9 times longer (n = 8).

Cephalothorax bulbous bearing pair of antennary swellings anteriorly on ventral surface (Fig. 1B, as; Fig. 2B, C); frontal margin of antennary swellings typically just visible in dorsal view (Fig. 1A, 2A). Paired posterior swellings on ventral surface of cephalothorax clearly visible in dorsal view (Fig. 1A). Two additional paired swellings present on ventral surface of cephalothorax, between antennary and posterior swellings (Fig. 1B, ps): smaller maxillary swellings (Fig. 1B, ms) and larger ventrolateral swellings (Fig. 1B, vls). Paired maxillary and ventrolateral swellings confluent at base and separated, along ventral midline, by narrow raised central ridge (Fig. 1B, cr).

Antennule cylindrical, indistinctly segmented; setation not observed. Antenna (Fig. 1C) biramous; protopodal part unarmed; exopod unsegmented, bulbous, shorter than endopod; armed with inner and outer setae subapically; ornamented with minute denticles on rounded apex; endopod 2-segmented, proximal segment ornamented with spinules distally; distal margin of terminal segment bearing robust outer hook, 2 stout naked setae, and 1 minute seta; inner extremity of margin slightly inflated and ornamented with minute denticles. Mouth tube not observed clearly. Mandible (Fig. 1D) stylet-like, forming slender blade with 9 marginal teeth increasing in size towards tip. Maxillule (Fig. 1E) slender with 1 small and 2 large setae around apex and 2 small setae on proximally located outer lobe. Maxilla (Fig. 1B, mx) comprising small digitiform process carried posteriorly on maxillary swelling. Maxillipeds located posterior to oral region and anterior to maxillary swellings; subchelate, each with broad protopodal corpus expanded into strongly denticulate myxal process opposing tip of subchela; subchela armed with blunt-tipped seta about midway along inner margin (Fig. 1F).

Male unknown.

Etymology. The shape of the head of this species resembles that of the character Squidward from the cartoon Sponge Bob. The new name, *squidwardi*, alludes to this resemblance.

### Remarks

The general body shape and body proportions of the transformed adult female of the new species are similar to several other congeneric species. Only two species, *T. australis* and *T. versicolor* (Wilson, 1919), have an unequivocal demarcation between the neck and the trunk (Kabata, 1993; Dippenaar, 2018), marked by the abrupt expansion of the trunk. In all other species the transition from neck to trunk is more or less gradual as in the new species. In three other species, *T. asymboli*, *T. triakis* (Castro Romero, 2001) and *T. beatricae* Dippenaar, 2018, the trunk is reasonably clearly defined and is short (less than half as long as the neck) (Castro Romero, 2001; Turner et al., 2003; Dippenaar, 2018). In the new species and remaining known species, the trunk is more than half the length of the trunk. In *T. ferox* (Wilson, 1919), as in *T. beatricae*, the posterior processes are distinctly longer than the trunk whereas in the new species these processes are shorter than the trunk. Distinguishing the new species from the remaining six valid species relies primarily on the number and configuration of the lobes on the cephalothorax, as well as the length of the posterior processes relative to the trunk.

*Tripaphylus vassierei* (Delamare Deboutteville & Nuñes-Ruivo, 1954) has a complex array of cephalothoracic swellings. In particular the paired antennary swellings are each composed of about five fused lobes forming an almost triangular protuberance (Dippenaar, 2018). In addition, the posterior processes of *T. vassierei* are about as long as the trunk. Both these character states serve to distinguish *T. vassierei* from the new species which has simple antennary swellings and posterior processes shorter than the trunk. *Tripaphylus lewisi* Dippenaar, 2018 also has a complex arrangement of cephalothoracic swellings, with the maxillary swellings being trilobate, but this species is distinctive in having the trunk longer than the neck, unlike the new species. In *T. benzi* Dippenaar, 2018 and *T. hoi* Dippenaar, 2018 the cephalothorax bears paired antennary and maxillary swellings (which may appear bilobate) but neither possesses well developed paired posterior swellings (Dippenaar, 2018). The type species, *T. musteli* (van Beneden, 1851), also lacks posterior swellings although it does possess paired lateral swellings (Benz & Boxshall, 2017). *Tripaphylus hemigalei* Kirtisinghe, 1964 is perhaps the least well known of the valid species but can readily be distinguished from the new species by the possession of paired lateral horn-like processes on the cephalothorax and by the shape of the cephalothorax, described by Kirtisinghe (1964) as one and a half times as wide as long. The new species lacks lateral horn-like lobes visible in dorsal view, and has a cephalothorax more than twice as wide as long.

The new species appears to be most closely related to *T. elongatus* (Wilson, 1922); both species have a similar array of antennary, maxillary and posterior swellings, both have the neck longer than the trunk (although the distinction between neck and trunk is not well marked in either), and the posterior processes are shorter than the trunk in both. There are, however, significant differences in the relative development of the cephalothoracic swellings. In *T. elongatus* the paired maxillary swellings are attached to a “larger central swelling consisting of 2 lateral knobs and 1 median knob” (Dippenaar, 2018: 175) whereas in the new species each of the small maxillary swellings is fused basally to a larger, more medially located ventrolateral swelling (vls) which are separated in the midline by a narrow central ridge (Fig. 1B, cr). The large fused central swelling of *T. elongatus* (see Dippenaar, 2018: Fig. 2A) serves as a diagnostic feature for this species and its absence in the new species allows the two species to be readily separated.

***Tripaphylus dippenaarae*** **new species**

Type Material.

Holotype female, intact with one missing egg sac, from *Rhizoprionodon acutus* (Rüppell, 1837) (RHIACU 43, collected 08 Nov 2018; -9.86694, 135.6608; Male 762 mm TL) MAGNT Reg. No. NTM Cr0179497; paratype female intact but lacking egg sacs, from *R. acutus* (RHIACU 65, collected 12 Nov 2018; -10.085, 135.9778; Male 800 mm TL), Reg. No. NHMUK 2022.178; headless paratype female, trunk bearing paired egg sacs, from *R. acutus* (RHIACU 87, collected 05 Dec 2018; -9.82, 135.49; Male 739 mm TL), NTM Cr0179498; headless paratype female, trunk bearing paired egg sacs, from *R. acutus* (RHIACU 91, collected 15 Feb 2019; -10.1167, 136.317; Female 804 mm TL), NTM Cr0179499.

LSID urn:lsid:zoobank.org:act:C53693BF-0A95-4CAB-90EF-9096D0745A32

### Description (Figs 3, 4)

**Fig. 3:**
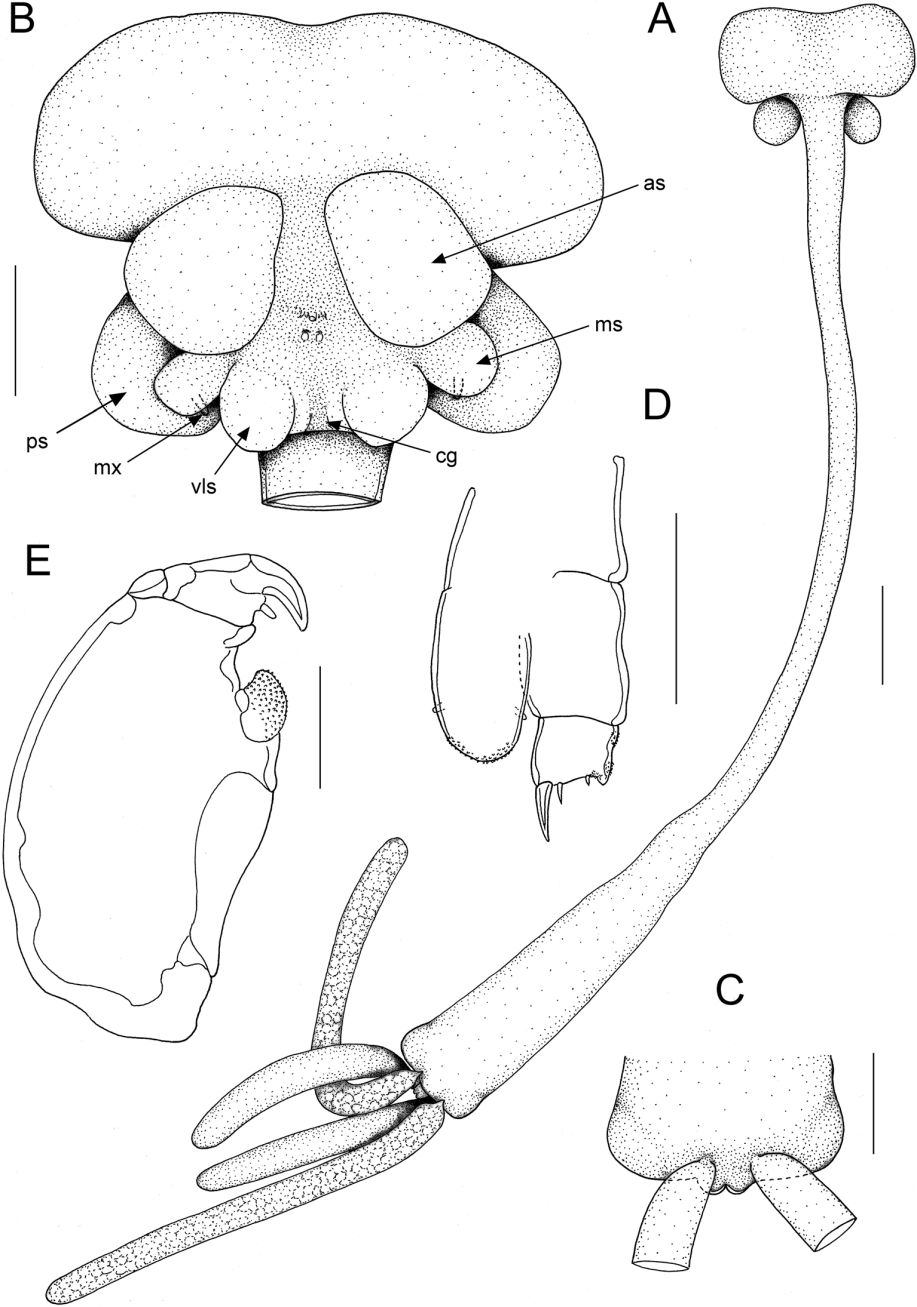
*Tripaphylus dippenaarae* **n. sp.** adult female. A, habitus, dorsal; B, cephalothorax, ventral; C, Posterior extremity of trunk and minute abdomen, ventral; D, antenna; E, maxilliped. Scale bars: A, 2 mm, B-C, 1 mm, D-E, 50 μm. Abbreviations: as = antennary swelling, cg = central groove, ms = maxillary swelling, mx = maxilla, ps = posterior swelling, vls = ventrolateral swelling

**Fig. 4:**
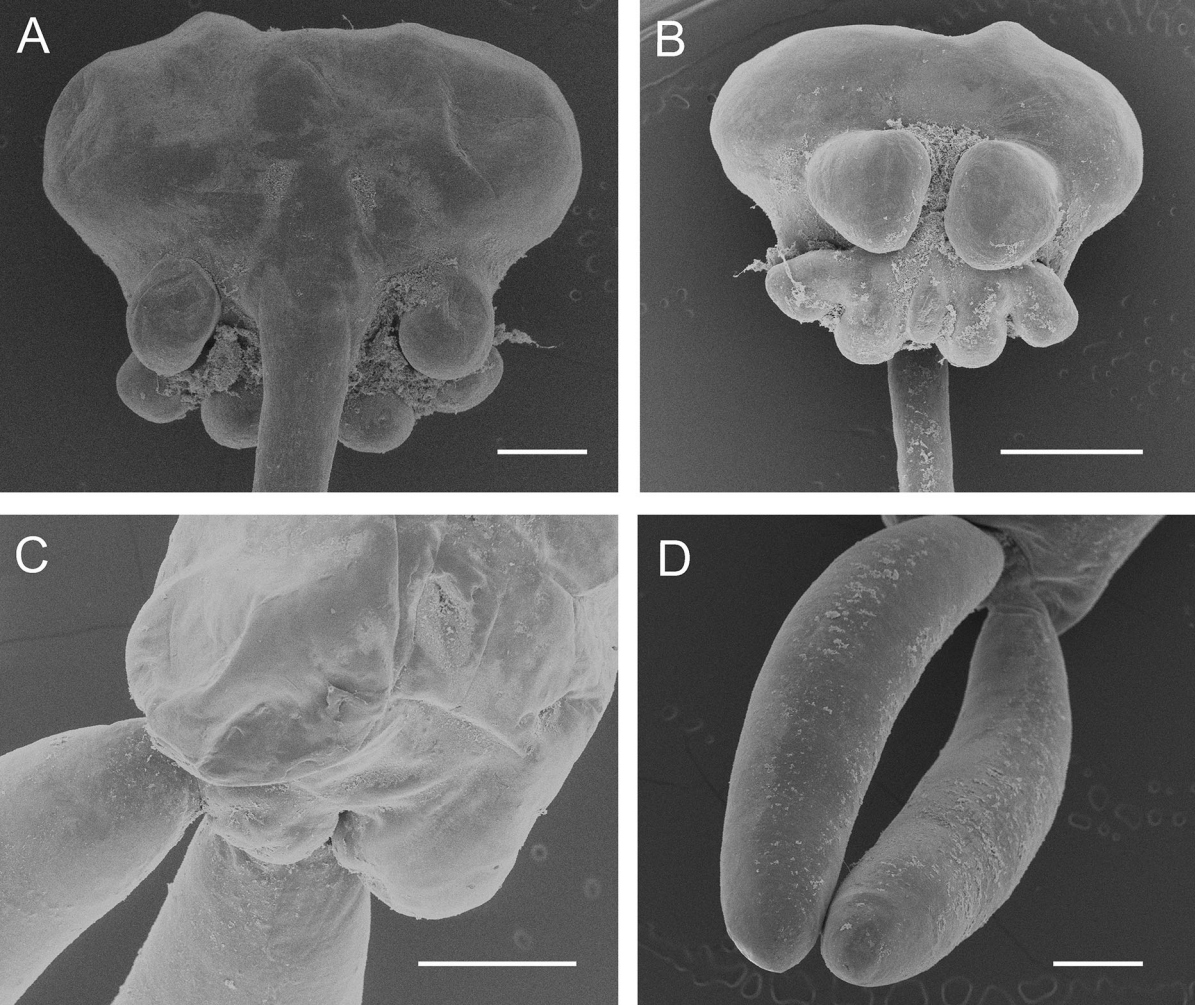
Scanning electron micrographs of *Tripaphylus dippenaarae* **n. sp.** adult female. A, cephalothorax, dorsal; B, cephalothorax, ventral; C, posterior extremity of trunk showing abdomen and origins of posterior processes; D, posterior processes. Scale bars A, C-D, 500 μm, B, 1.0 mm

Transformed adult female. Body (Fig. 3A) comprising cephalothorax, neck and trunk incorporating minute abdomen and bearing large paired posterior processes (Fig. 4D). Measurements of holotype female: total length measured from mid frontal margin of cephalothorax to posterior margin of bilobed anal prominence (excluding posterior processes) 25.4 mm; cephalothorax length 1.7 mm, width 4.1 mm; neck length about 15.0 mm, trunk length about 8.8 mm [neck + trunk combined length 23.7 mm], maximum trunk width 2.0 mm; posterior processes length 5.2 mm, width 0.6 mm; egg sac length 9.2 mm. Total body length of intact paratype 21.7 mm.

Neck slender, cylindrical with smooth margin; expanded anteriorly at junction with cephalothorax; expanding gradually posteriorly to merge with trunk. Neck longer than trunk, mean 1.75 times, with a range of 1.70 to 1.80 times longer (n = 2). Trunk about 4.4 times longer than wide, slightly compressed dorsoventrally and increasing in width towards maximum at posterior end; bearing paired lateral swellings near posterior end. Abdomen incorporated into trunk; bilobed posteriorly (Fig. 3C, 4C). Trunk bearing paired posterior processes ventrally; each process cylindrical, blunt tipped, and about 8.7 times longer than wide. Egg sacs multiseriate, originating from dorsally located oviduct openings near posterior margin of trunk; typically about twice as long as posterior processes, ranging from 1.8 to 2.3 times longer (n = 2).

Cephalothorax bulbous bearing pair of antennary swellings anteriorly on ventral surface (Fig. 3B, as; Fig. 4B). Paired posterior swellings on ventral surface of cephalothorax clearly visible in dorsal view (Fig. 3A, B, ps; Fig. 4A). Two additional paired swellings present on ventral surface of cephalothorax, posterior to antennary swellings (Fig. 3B): smaller and laterally located maxillary swellings (Fig. 3B, ms) and slightly larger medially located ventrolateral swellings (Fig. 3B, vls). Ventrolateral swellings with conspicuously red-pigmented tips. Paired ventrolateral swellings separated along ventral midline by well-defined median groove along centre line (Fig. 3B, cg).

Antennule not observed. Antenna (Fig. 3D) biramous; protopodal part unarmed; exopod unsegmented, bulbous, shorter than endopod; armed with inner and outer setae subapically; ornamented with minute denticles on rounded apex; endopod 2-segmented, proximal segment unornamented; distal margin of terminal segment bearing robust outer hook, 2 slender naked setae, and minute seta; inner extremity of margin slightly inflated and ornamented with minute denticles. Mouth tube and oral appendages not observed. Maxilla (Fig. 3B, mx) comprising small digitiform process carried posteriorly on maxillary swelling. Maxillipeds located posterior to oral region and anterior to maxillary swellings; subchelate, each with broad protopodal corpus expanded into strongly denticulate myxal process opposing tip of subchela; subchela armed with blunt-tipped seta about midway along inner margin (Fig. 3E).

Male unknown.

Etymology. The species name, dippenaarae, honours Susan Dippenaar for her major contributions to our knowledge of the diversity of *Tripaphylus*.

### Remarks

This new species is similar to *T. elongatus* and *T. squidwardi* **n. sp.** All three species have a similar array of antennary, maxillary and posterior swellings on the cephalothorax, the neck is longer than the trunk (although the distinction between neck and trunk is not well marked), and the posterior processes are shorter than the trunk. The new species differs from *T. elongatus* in the relative development of the cephalothoracic swellings. In *T. elongatus* the paired maxillary swellings are attached to a “larger central swelling consisting of 2 lateral knobs and 1 median knob” (Dippenaar, 2018) whereas in the new species each of the small maxillary swellings arises immediately adjacent to a slightly larger, more medially located, ventrolateral swelling. These swellings are separated in the midline by a narrow central groove (Fig. 3B, cg). The large fused central swelling of *T. elongatus* (see Dippenaar, 2018: Fig. 2A) is absent in the new species.

The new species is most similar to *T. squidwardi* **n. sp.** but these two species differ slightly in body proportions: in *T. squidwardi* **n. sp.** the neck is a mean of 1.48 times longer than the trunk whereas in *T. dippenaarae* **n. sp.** the neck is a mean of 1.75 times longer than the trunk. However, the range of values for *T. dippenaarae* **n. sp.** falls within the range found in *T. squidwardi* **n. sp.** This plus the difficulty in determining the precise boundary between neck and trunk makes this difference unreliable for species discrimination.

There are additional differences between these two new species in the precise arrangement of the lobes on the ventral surface of the cephalothorax. The most striking difference is the relative size of the maxillary swellings and the ventrolateral swellings: in *T. squidwardi* **n. sp.** the maxillary swellings are about one third of the size of the ventrolateral swellings whereas in *T. dippenaarae* **n. sp.** the maxillary swellings are only slightly smaller than the ventrolateral swellings. In addition, the paired ventrolateral swellings are separated along ventral midline by a central ridge in *T. squidwardi* **n. sp.** but by a well-defined median groove in *T. dippenaarae* **n. sp.**

## Data Availability

Type material is deposited in collections of Museum and Art Gallery, Northern Territory and Natural History Museum, London (see text for details) and is available for study. Collections data are available in museum registers.
